# Drying Strawberry Slices: A Comparative Study of Electrohydrodynamic, Hot Air, and Electrohydrodynamic‐Hot Air Techniques

**DOI:** 10.1002/fsn3.4541

**Published:** 2024-10-20

**Authors:** Ahmet Polat

**Affiliations:** ^1^ Department of Biosystems Engineering, Faculty of Agriculture Bursa Uludag University Bursa Turkey

**Keywords:** color, drying times, electrohydrodynamic, microstructure, modeling, strawberry

## Abstract

The investigation encompassed an examination of the drying durations, modeling, and quality attributes (color, rehydration capacity, microstructural features, total soluble solid [TSS], and pH values) of strawberry slices subjected to diverse drying methodologies, namely electrohydrodynamic (EHD), EHD‐hot air, and hot air processes. Furthermore, 10 distinct thin‐layer drying models were applied, and their goodness‐of‐fit was assessed to identify the most suitable model for the drying process. This analysis encompassed applying two distinct temperatures (50°C and 55°C), and voltage levels (20 and 30 kV). The EHD method yielded the lengthiest drying durations for the strawberry samples, with hot air and EHD‐hot air drying techniques subsequent in descending order of drying time. The outcomes of the modeling analyses demonstrated that the thin‐layer drying behaviors of used drying methods were best described by the Midilli et al. and Wang and Singh models in terms of their goodness‐of‐fit. A decline in the L* values was noted with the elevation of temperature in the hot air drying method and with the escalation of voltage in the EHD drying. The minimum a* value was detected in the hot air drying method conducted at 55°C. The rehydration capacity of strawberry samples subjected to the EHD and EHD‐hot air combination drying methods (except 20 kV‐55°C) did not exhibit any statistically significant variation in response to different voltage settings. Although the structure of strawberry samples dried with 20 kV application was observed as smoother, cracks occurred on the product surface in other drying applications. In hot air and EHD methods, varying temperature and volt applications did not show a significant effect on TSS and pH values. As a result, it has been seen that EHD technology, which is a promising drying method used in this study, is a suitable method in terms of processing efficiency and consumer acceptability of dried strawberry products.

## Graphical abstract

Increasing voltage and temperature reduced the drying time of the products. Electrohydrodynamic (EHD)‐dried samples exhibited lower Δ*E* values compared with hot air and EHD‐hot air combination methods. It is concluded that EHD technology can work in harmony with hot air and can be an alternative method to industrial‐scale drying devices.

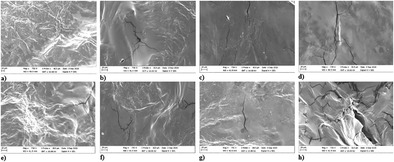

## 1 Introduction

The strawberry (Fragaria × ananassa), a member of the Rosaceae family, stands as one of the most widely consumed fruits globally (Yan et al. 2018). Global production in 2021 reached a staggering 9,175,384.43 tons, with China leading as the top producer at 3,380,478.19 tons, followed closely by the United States, Turkiye, and Mexico (FAO 2021). Strawberry is shown among the berry fruits that contain both flavor and nutritional value and important bioactive components (especially vitamin C). Studies have reported that vitamin C is effective in preventing obesity, type 2 diabetes, cancer, inflammation, and cardiovascular diseases (Gamboa‐Santos et al. 2014). Strawberry fruit is generally consumed fresh. Due to its elevated respiration rate, susceptibility to weight loss, and propensity for microbial contamination, this product is classified among the delicate and perishable items. Therefore, it is processed and transformed into different forms such as dried, jelly concentrated jam, fruit juice, and bakery products to ensure consumption during the season (El‐Beltagy, Gamea, and Essa 2007; de Bruijn and Bórquez 2014). Among these procedures, drying stands out as one of the oldest preservation techniques. This method not only facilitates the creation of product variations and novel designs but also prolongs shelf life and decreases the bulk of agricultural commodities (Dinani, Hamdami, Shahedi, and Havet 2015). For numerous years, the conventional practice of employing hot air in convective drying has remained a prominent technique for processing agricultural goods. Despite its extensive adoption across the drying sector, this method ranks among the most energy‐intensive processes. Statistics reveal that about a quarter (25%) of industrial energy usage in developed nations is allocated to drying operations (Gamboa‐Santos et al. 2014). Furthermore, the consistent exposure to elevated temperatures through hot air, which exerts an impact on product quality, including nutrient content, color, flavor, taste, and shape, stands out as a significant drawback associated with this approach (Szadzińska, Kowalski, and Stasiak 2016). Novel drying technologies have been innovated with the primary objectives of mitigating quality degradation in products and enhancing energy efficiency in the drying process. One of these is electrohydrodynamic (EHD) drying, which is a heat‐sensitive method that has been investigated in depth recently. At the basis of the EHD technique lies a secondary airflow known as the corona wind. This flow is induced by the application of high voltage to a curved electrode, typically in the form of a needle, wire, or pin. Under the impact of high voltage, the air surrounding the pointed electrode undergoes ionization. Ions sharing the same polarity as the pointed electrode are swiftly propelled toward the grounded electrode. High‐velocity ions strike uncharged air molecules along their path, transmitting their movements and creating an ion flow called the corona wind. The corona wind disturbs the boundary layer of the biological material, consequently enhancing the rate of moisture evaporation (Dinani, Hamdami, Shahedi, Havet, and Queveau 2015). Similar to numerous drying methodologies, the efficiency of drying diminishes as the duration of the process extends. In the case of EHD drying, there is a reported potential advantage in integrating traditional drying techniques to expedite the process and facilitate moisture migration to the surface. Given this, innovative equipment has been developed, harnessing the advantages of both approaches (Dinani et al. 2014). Within this study, strawberry samples underwent drying via the methods of EHD, hot air, and a combination of EHD‐hot air. The study delved into the impact of these distinct techniques on drying durations, modeling, coloration, rehydration capabilities, and microstructural characteristics of the strawberry samples.

## 2 Materials and Methods

### 2.1 Fresh Product

Fresh samples of strawberry (Fragaria × ananassa) were procured from a local market situated in Bursa. The harvest place of the products is the İnegöl district of Bursa. Rotten and deformed products were separated, and the remaining part was placed in plastic bags so that the product would not be damaged. The items were kept under storage conditions of 4 ± 0.5°C until the experiments reached completion. To ascertain the initial moisture content of the strawberry product, a randomly selected subset of items was subjected to an oven set at 70°C for a period of 24 h. As a result of the calculations, the initial humidity was determined as 7.10 (d.b.). The samples were sliced using a cutting tool to initiate the drying procedure. Strawberry dimensions were found to be 24.10 ± 3.84 mm in diameter and 2.36 ± 0.23 mm in thickness with the help of a caliper. Each experiment utilized a sample weighing 130 ± 0.01 g.

### 2.2 Drying Process

The custom‐designed EHD‐convective dryer was employed to dry the sliced strawberry samples until they achieved a final moisture content of 0.1 (d.b.). The drying process involved the utilization of two distinct voltage levels (20 and 30 kV) and two varying temperature settings (50°C and 55°C). Every drying instance was executed under an air velocity of 1.5 m/s. Using these values, eight different drying applications (20 kV, 30 kV, 50°C, 55°C 20 kV‐50°C, 20 kV‐55°C, 30 kV‐50°C, and 30 kV‐55°C) were arranged. The EHD drying method was facilitated using a wire system. In the EHD wire system, six wires (0.4 mm diameter) were positioned 5 cm apart (Dinani and Havet 2015). In the drying process, a stainless steel plate measuring 26.5 × 34.5 cm was used. A gap of 30 mm was established between the dried product and the wire electrode. Moisture loss during the drying process was gauged every 30 min using a precision scale positioned beneath the dryer, accurate to 0.01. The drying procedures were conducted within controlled laboratory settings at a temperature of 27.1 ± 0.1°C and humidity of 23.8 ± 0.1%. The experiments were replicated thrice for each condition.

### 2.3 Thin‐Layer Modeling

The experimental outcomes from the dried strawberry samples were matched against 10 distinct thin‐layer models enumerated in Table 1. The model coefficients (*a*, *b*, *c*, *g*, *n*), (*k*, *k*_0_, and *k*_1_), and *t* within the equations correspond to the drying rate constant (1/min), drying time, and time, respectively (Therdthai and Zhou 2009). The moisture ratio of the drying strawberry samples was computed employing Equation (1).

(1) $$\mathrm{MR}\;=\;\frac{M_{t}-M_{e}}{M_{o}-M_{e}}$$

**TABLE 1:** Thin‐layer drying models used for mathematical modeling of strawberry samples.

No	Model Name	Model	Reference
1	Henderson and Pabis	$\mathrm{MR}=a\exp\left(-\mathrm{kt}\right)$	Demiray and Tulek (2014)
2	Newton	$\mathrm{MR}=\exp\left(-\mathrm{kt}\right)$	Taskin (2020)
3	Page	$\mathrm{MR}=\exp\left(-{\mathrm{kt}}^{n}\right)$	Murthy and Manohar (2014)
4	Logarithmic	$\mathrm{MR}=a\exp\left(-\mathrm{kt}\right)+c$	Amiri Chayjan and Shadidi (2014)
5	Two Term	$\mathrm{MR}=a\exp\left(-k_{0}t\right)+b\exp\left(-k_{1}t\right)$	Bhattacharya, Srivastav, and Mishra (2015)
6	Two Term Exponential	$\mathrm{MR}=a\exp\left(-\mathrm{kt}\right)+\left(1-a\right)\exp\left(-\mathrm{kat}\right)$	Evin (2011)
7	Wang and Singh	$\mathrm{MR}=1+\mathrm{at}+{\mathrm{bt}}^{2}$	Belghith, Azzouz, and ElCafsi (2016)
8	Diffusion Approach	$\mathrm{MR}=a\exp\left(-\mathrm{kt}\right)+\left(1-a\right)\exp\left(-\mathrm{kbt}\right)$	Taşkın, İzli, and İzli (2018)
9	Verma et al.	$\mathrm{MR}=a\exp\left(-\mathrm{kt}\right)+\left(1-a\right)\exp\left(-\mathrm{gt}\right)$	Faal, Tavakoli, and Ghobadian (2015)
10	Midilli et al.	$\mathrm{MR}=a\exp\left(-{\mathrm{kt}}^{n}\right)+\mathrm{bt}$	Midilli, Kucuk, and Yapar (2002)

In this context, the variables $M_{t}$, $M_{o}$, and $M_{e}$ denote the moisture content at any time, the initial moisture content, and the equilibrium moisture content (kg water/kg dry matter), respectively. Certain researchers have transformed Equation (1) into Equation (2) due to the insignificantly small magnitude of the $M_{e}$ value in comparison with the $M_{t}$ and $M_{o}$ values (Erbay and Icier 2010).

(2) $$\mathrm{MR}\;=\;\frac{M_{t}}{M_{o}}$$

### 2.4 Determination of Color Values

The surface color attributes of strawberry samples, subjected to different drying methods, were assessed using a colorimeter (MSEZ‐4500 L, HunterLab, USA). Prior to recording the color values, the instrument underwent calibration employing black and white plates. Post‐calibration, the samples were positioned within a glass petri dish, and color values were obtained from five distinct points. The L*, a*, and b* values displayed on the device screen represent lightness/darkness, redness/greenness, and yellowness/blueness, respectively. Chroma (*C*), a measure of color intensity, Hue angle (*α°*), indicating diminished browning, and Δ*E*, representing the overall color disparity, were computed using the subsequent formulas (Dehghannya, Gorbani, and Ghanbarzadeh 2017; Li et al. 2017).

(3) $$C\;=\;\sqrt{\left(a^{2}+b^{2}\right)}$$

(4) $$\alpha \;=\;\tan^{-1}\left(\frac{b}{a}\right)$$

(5)

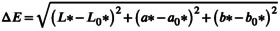




The formulas incorporate the L*, a*, and b* values representing the color characteristics of the dried samples, whereas the *L*_0_*, *a*_0_*, and *b*_0_* values signify the color attributes of the fresh samples.

### 2.5 Determination of Rehydration Capacity

Rehydration capacity assessments for the strawberry samples were conducted by immersing the dried samples in a beaker at a solid–liquid ratio of 1:50 for a duration of 14 h (de Jesus Junqueira et al. 2017). Subsequently, the liquid in the beaker was decanted, and any residual moisture on the rehydrated product was carefully absorbed using filter paper. The rehydrated products were then weighed on a precise scale and documented. The rehydration capacity values were computed using Equation (5) (Feng et al. 2021):

(6) $$\text{Rehydration capacity}\;=\;\frac{M_{2}-M_{1}}{M_{1}}$$

Here, *M*_1_ represents the weight of the dried sample and *M*_2_ signifies the weight of the rehydrated sample. The experiments were conducted in triplicate.

### 2.6 Microstructure Analysis

Surface microstructural alterations of strawberry samples subjected to various drying methods were examined using a scanning electron microscope (SEM) (EVO 40, Germany) (Xiao et al. 2009). Samples taken from certain parts of each product were positioned in holders and coated with gold palladium under vacuum conditions. After this process, the coated samples were placed in the SEM device, and their micrographs were taken.

### 2.7 Statistical Analysis

The data acquired from the experiments involving dried strawberry samples were imported into the MS‐Excel software. Analysis of the data, except for the modeling aspects, was conducted using the JMP software (Version 7.0; SAS Institute Inc., Cary, NC, USA). To fit the experimental data with the thin‐layer mathematical models specified in Table 1, a nonlinear regression analysis was executed using the MATLAB software package (MathWorks Inc., Natick, MA). The coefficient of determination (*R* ^2^), chi‐squared (*χ* ^2^), and root mean square error (RMSE) statistical parameters were used to determine the model that best explains the drying behavior of the strawberry product. The calculation of the RMSE and chi‐squared (*χ* ^2^) values was achieved through Equations (7) and (8), respectively (Doymaz, Kipcak, and Piskin 2015):

(7) $$\text{RMSE}\;=\;\sqrt{\frac{\sum_{İ=1}^{n}{\left({\mathrm{MR}}_{\mathrm{pre},i}-{\mathrm{MR}}_{\exp,i}\right)}^{2}}{N}}$$

(8) $$\chi^{2}\;=\;\frac{\sum_{İ=1}^{N}{\left({\mathrm{MR}}_{\exp,i}-{\mathrm{MR}}_{\mathrm{pre},i}\right)}^{2}}{N-z}$$

In the equation, ${\mathrm{MR}}_{\mathrm{pre},i}$ and ${\mathrm{MR}}_{\exp,i}$ denote the estimated moisture ratio for test number *i*, the experimental moisture ratio for test number *i*, respectively. Additionally, N stands for the count of observed experimental data, whereas z representing the count of independent variables within the model.

## 3 Results and Discussion

### 3.1 Drying Curves of Strawberry Slices

**FIGURE 1:**
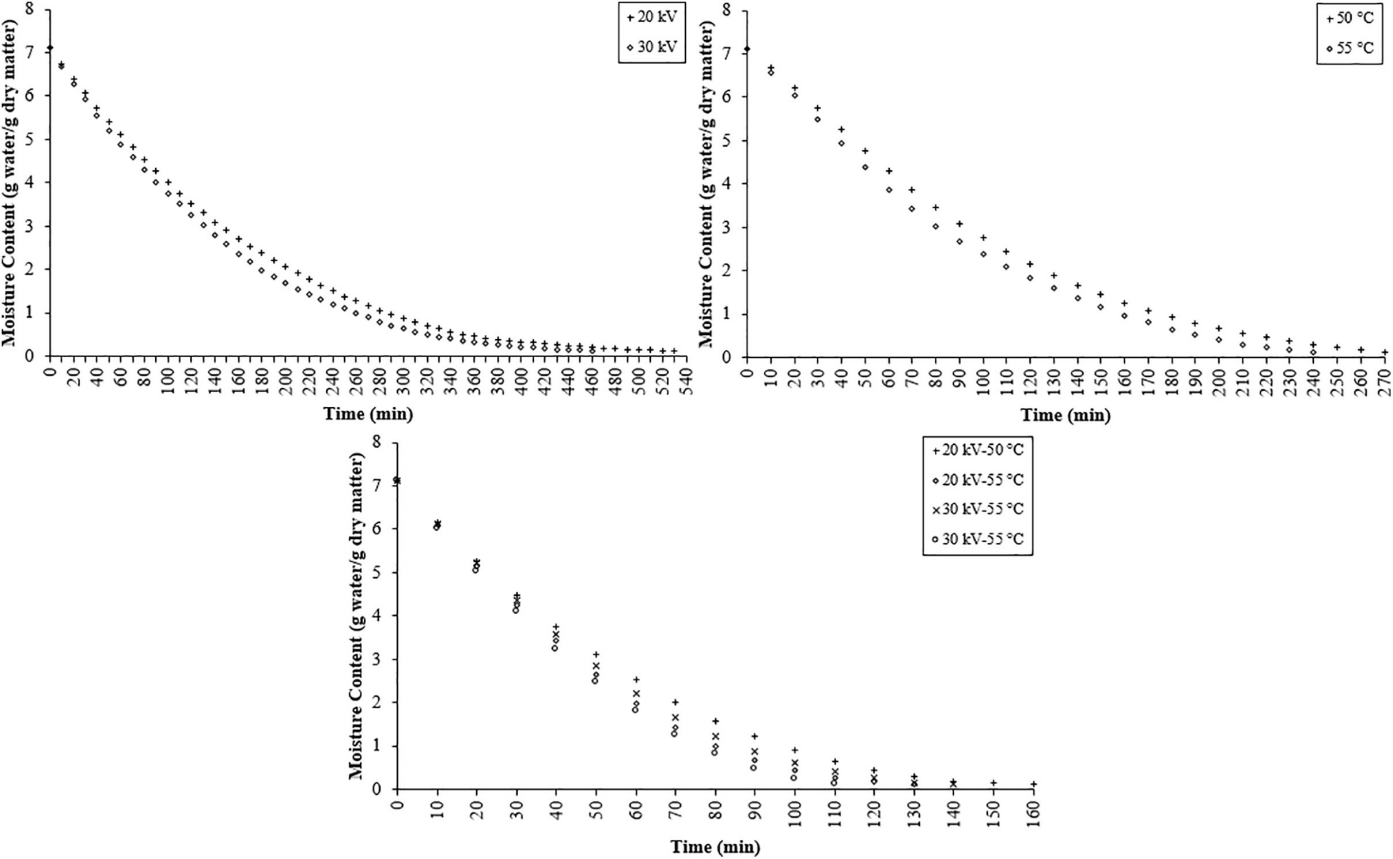
Variation of moisture content of strawberry samples dried under different drying conditions over time.

Figure 1 illustrates the variation of moisture content of strawberry samples dried under different drying conditions (EHD, EHD‐hot air, and hot air) over time. The figure reveals a reduction in product moisture content as drying time progresses. Comparing the methods, it is evident that EHD‐dried samples took longer to attain the final moisture content in contrast to those dried using hot air. A similar observation was made by Bai et al. (2013) in their investigation of sea cucumber drying. For the strawberry samples, drying durations at 50°C and 55°C were determined as 270 and 240 min, respectively. The elevated temperatures in the drying process expedited heat transfer between the product and the heat source, resulting in accelerated moisture removal and reduced drying times (Beigi 2016). These findings align with previous research on agricultural product drying (Kumar, Sarkar, and Sharma 2012; Tunde‐Akintunde and Ogunlakin 2013). Drying durations for the 20 kV‐55°C, 30 kV‐50°C, and 30 kV‐55°C applications were notably shorter by 18.75%, 12.5%, and 31.25%, respectively, in comparison with the 20 kV‐50°C applications. Within the EHD combined method, an increase in temperature and voltage resulted in a decrease in drying time. Specifically, samples dried with a 20 kV application required 530 min, whereas those dried with a 30 kV application took 460 min. Similar to our study, Pirnazari, Esehaghbeygi, and Sadeghi (2016) observed a decline in drying time with heightened electric field strength in a study on bananas.

### 3.2 Modeling of Drying Curves

**FIGURE 2:**
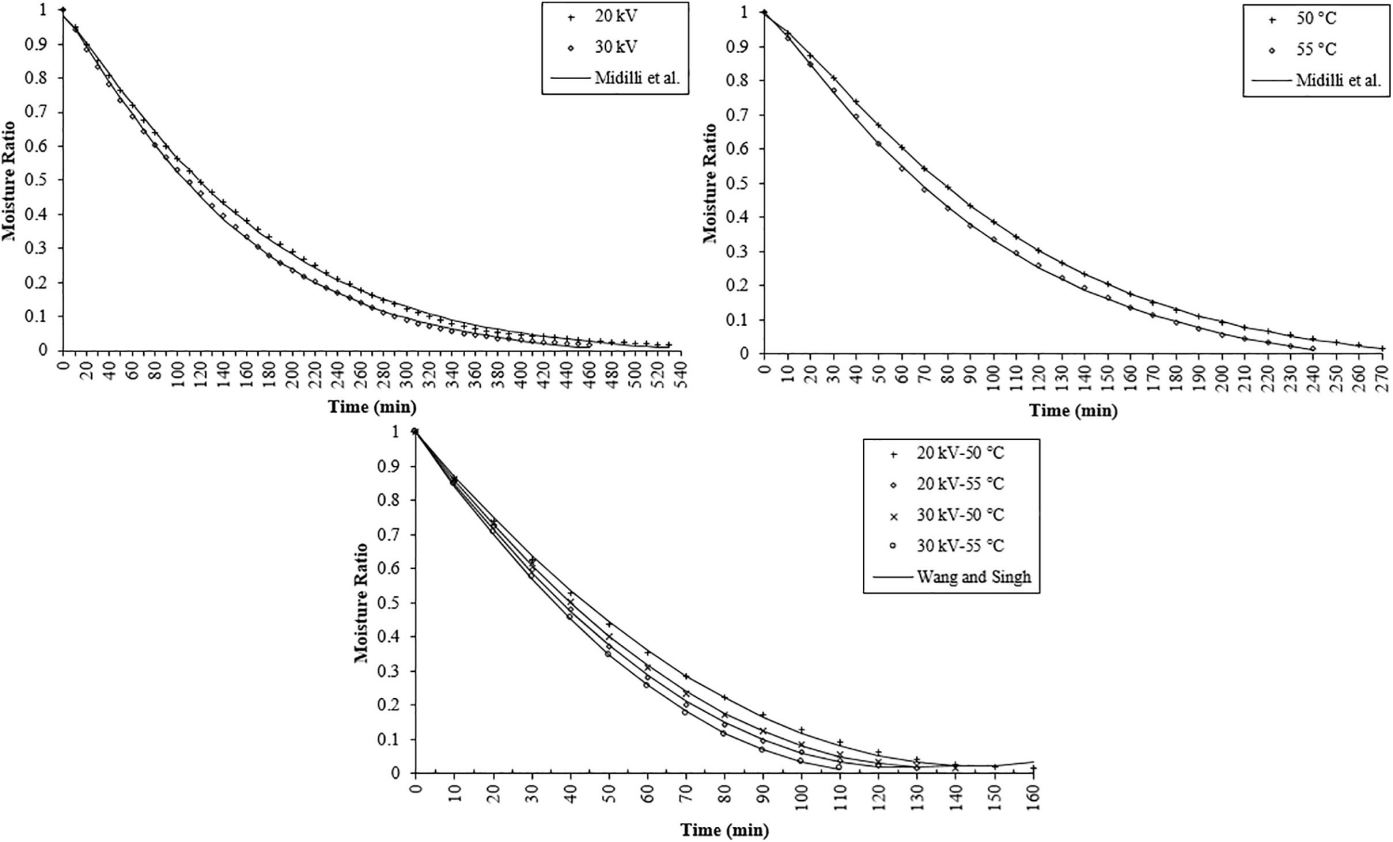
Variation of experimental moisture content of strawberry samples dried under different drying conditions and estimated moisture content obtained from Midilli et al. and Wang and Singh models.

The investigation encompassed the variation of moisture ratio values throughout the drying process of strawberry slices utilizing EHD, EHD‐hot air, and hot air methods. This variation was examined concerning drying times through the application of 10 thin‐layer drying models outlined in Table 1. The coefficients of the employed models across all drying conditions were assessed by computing statistical parameters *R* ^2^, *χ* ^2^, and RMSE. These findings are presented in Tables 2 and 3. Within these tables, *R* ^2^ values ranged from 0.9680 to 0.9999, *χ* ^2^ values ranged from 0.0309 × 10^−4^ to 35.8827 × 10^−4^, and RMSE values ranged from 0.0017 to 0.0599. For model selection, the criteria involved favoring the highest *R* ^2^ values alongside the lowest RMSE and *χ* ^2^ values. A thorough examination of Table 2 revealed that the drying behavior of strawberry samples subjected to EHD and hot air drying methods aligned well with the model proposed by Midilli et al. Table 3 demonstrates that, for the EHD‐hot air combined method, the Wang and Singh model consistently displayed the highest *R* ^2^ values and the lowest RMSE and *χ* ^2^ values across all investigated cases. As a result, the Wang and Singh model was selected as the most suitable representation for the thin‐layer drying process of strawberries. Figure 2 illustrates the variation of experimental moisture content of strawberry samples dried under different drying conditions and estimated moisture content obtained from Midilli et al. and Wang and Singh models. The figure depicts a remarkable degree of similarity between the curves, indicating that the chosen models can adequately predict the moisture ratio of strawberry slices at various time points during the EHD, hot air, and EHD‐hot air drying processes. These findings align with previous research studies. Takougnadi, Boroze, and Azouma (2018), Zhang et al. (2018), and Krzykowski et al. (2020) found a good fit of experimental data to the Midilli et al. model in their respective drying investigations, which resonates with our research. Similarly, our findings for the Wang and Singh model are in line with the results reported by Doymaz (2008), Wilson et al. (2012), and Omolola, Jideani, and Kapila (2014).

**TABLE 2:** Statistical analysis results from models used for strawberry slices dried under EHD and hot air drying conditions.

No.	20 kV				30 kV				50°C				55°C			
	Model Katsayıları	*R* ^2^	RMSE	*χ* ^2^ (10^−4^)	Model Katsayıları	*R* ^2^	RMSE	*χ* ^2^ (10^−4^)	Model Katsayıları	*R* ^2^	RMSE	*χ* ^2^ (10^−4^)	Model Katsayıları	*R* ^2^	RMSE	*χ* ^2^ (10^−4^)
1	*a* = 1.052 *k* = 0.006687	0.9943	0.0217	4.7164	*a* = 1.048 *k* = 0.00742	0.9942	0.0221	4.8703	*a* = 1.079 *k* = 0.01078	0.9880	0.0339	11.4921	*a* = 1.067 *k* = 0.01204	0.9897	0.0313	9.7701
2	*k* = 0.006368	0.9917	0.0263	6.8972	*k* = 0.007091	0.9919	0.0260	6.7834	*k* = 0.009998	0.9811	0.0425	18.0275	*k* = 0.0113	0.9847	0.0380	14.4004
3	*k* = 0.002639 *n* = 1.168	0.9991	0.0089	0.7909	*k* = 0.003061 *n* = 1.163	0.9990	0.0093	0.8662	*k* = 0.002605 *n* = 1.283	0.9995	0.0069	0.4840	*k* = 0.003707 *n* = 1.241	0.9988	0.0109	1.1795
4	*a* = 1.081 *k* = 0.005743 *c* = −0.05605	0.9983	0.0118	1.3859	*a* = 1.083 *k* = 0.006273 *c* = −0.06336	0.9987	0.0105	1.1018	*a* = 1.174 *k* = 0.008035 *c* = −0.1352	0.9977	0.0148	2.1971	*a* = 1.159 *k* = 0.009029 *c* = −0.131	0.9988	0.0106	1.1162
5	*a* = 0.5268 *k*_o_ = 0.006675 *b* = 0.5268 *k*_1_ = 0.00672	0.9941	0.0222	4.9078	*a* = 0.5242 *k*_o_ = 0.007424 *b* = 0.5242 *k*_1_ = 0.007424	0.9939	0.0226	5.0970	*a* = 0.9545 *k*_o_ = 0.01077 *b* = 0.1247 *k*_1_ = 0.01077	0.9870	0.0353	12.4493	*a* = 0.8814 *k*_o_ = 0.01204 *b* = 0.1851 *k*_1_ = 0.01204	0.9887	0.0327	10.7006
6	*a* = 1.691 *k* = 0.008359	0.9991	0.0087	0.7522	*a* = 1.684 *k* = 0.009278	0.9991	0.0088	0.7764	*a* = 1.002 *k* = 0.009998	0.9804	0.0433	18.7209	*a* = 1.787 *k* = 0.01573	0.9984	0.0124	1.5409
7	*a* = −0.004566 *b* = 0.00005304	0.9943	0.0218	4.7633	*a* = −0.005126 *b* = 0.000006723	0.9948	0.0208	4.3344	*a* = −0.007445 *b* = 0.00001433	0.9990	0.0099	0.9869	*a* = −0.008391 *b* = 0.00001823	0.9985	0.0117	1.3736
8	*a* = 5.499 *k* = 0.003946 *b* = 0.9017	0.9986	0.0110	1.2031	*a* = 0.6474 *k* = 0.007092 *b* = 0.9997	0.9916	0.0266	7.0917	*a* = 5.28 *k* = 0.004984 *b* = 0.8422	0.9968	0.0175	3.0733	*a* = 0.7501 *k* = 0.0113 *b* = 0.999	0.9834	0.0396	15.7095
9	*a* = 7.297 *k* = 0.009807 g = 0.01065	0.9993	0.0076	0.5739	*a* = 0.8821 *k* = 0.007091 g = 0.007088	0.9916	0.0266	7.0917	*a* = −2.567 *k* = 0.003958 g = 0.005191	0.9968	0.0176	3.0858	*a* = 0.0198 *k* = 0.01126 g = 0.0113	0.9834	0.0396	15.7096
10	*a* = 0.9846 *k* = 0.002549 *n* = 1.167 *b* = −0.00002448	0.9995	0.0067	0.4457	*a* = 0.9841 *k* = 0.003069 *n* = 1.154 *b* = −0.00003929	0.9996	0.0059	0.3494	*a* = 0.9963 *k* = 0.003046 *n* = 1.24 *b* = −0.00009402	0.9999	0.0017	0.0309	*a* = 1.001 *k* = 0.005053 *n* = 1.158 *b* = −0.0001814	0.9998	0.0042	0.1773

**TABLE 3:** Statistical analysis results from models used for strawberry slices dried under EHD‐hot air combined drying conditions.

No.	20 kV‐50°C				20 kV‐55°C				30 kV‐50°C				30 kV‐55°C			
	Model Katsayıları	*R* ^2^	RMSE	*χ* ^2^ (10^−4^)	Model Katsayıları	*R* ^2^	RMSE	*χ* ^2^ (10^−4^)	Model Katsayıları	*R* ^2^	RMSE	*χ* ^2^ (10^−4^)	Model Katsayıları	*R* ^2^	RMSE	*χ* ^2^ (10^−4^)
1	*a* = 1.054 *k* = 0.01931	0.9876	0.0355	12.6346	*a* = 1.066 *k* = 0.02254	0.9809	0.0459	21.0616	*a* = 1.063 *k* = 0.02124	0.9834	0.0422	17.7644	*a* = 1.062 *k* = 0.02378	0.9769	0.0509	25.8672
2	*k* = 0.01837	0.9849	0.0391	15.3148	*k* = 0.02123	0.9771	0.0503	25.3020	*k* = 0.02004	0.9798	0.0466	21.7129	*k* = 0.02244	0.9738	0.0542	29.3585
3	*k* = 0.006541 *n* = 1.247	0.9980	0.0143	2.0518	*k* = 0.005469 *n* = 1.338	0.9983	0.0138	1.9152	*k* = 0.005675 *n* = 1.31	0.9984	0.0130	1.6850	*k* = 0.005682 *n* = 1.349	0.9966	0.0195	3.8148
4	*a* = 1.141 *k* = 0.01465 *c* = −0.1215	0.9978	0.0148	2.2007	*a* = 1.195 *k* = 0.01595 *c* = −0.1668	0.9954	0.0225	5.0668	*a* = 1.177 *k* = 0.01541 *c* = −0.1503	0.9963	0.0199	3.9549	*a* = 1.282 *k* = 0.01471 *c* = −0.2643	0.9975	0.0168	2.8260
5	*a* = 0.5266 *k*_o_ = 0.0193 *b* = 0.5266 *k*_1_ = 0.0193	0.9856	0.0382	14.5782	*a* = 0.5328 *k*_o_ = 0.02254 *b* = 0.5328 *k*_1_ = 0.02254	0.9771	0.0503	25.2737	*a* = 0.9172 *k*_o_ = 0.02124 *b* = 0.1458 *k*_1_ = 0.02119	0.9804	0.0458	20.9942	*a* = 0.5308 *k*_o_ = 0.02378 *b* = 0.5308 *k*_1_ = 0.02378	0.9712	0.0569	32.3338
6	*a* = 1.005 *k* = 0.01837	0.9839	0.0404	16.3358	*a* = 1.871 *k* = 0.03077	0.9969	0.0186	3.4478	*a* = 1.002 *k* = 0.02004	0.9782	0.0484	23.3831	*a* = 1.871 *k* = 0.0327	0.9947	0.0244	5.9702
7	*a* = −0.01341 *b* = 0.00004601	0.9994	0.0079	0.6287	*a* = −0.01558 *b* = 0.00006177	0.9995	0.0073	0.5293	*a* = −0.01471 *b* = 0.00005523	0.9997	0.0053	0.2854	*a* = −0.01639 *b* = 0.00006724	0.9997	0.0055	0.2986
8	*a* = 0.8711 *k* = 0.01837 *b* = 1	0.9828	0.0418	17.5026	*a* = 0.7536 *k* = 0.02124 *b* = 0.9987	0.9729	0.0547	29.9024	*a* = 0.7763 *k* = 0.02004 *b* = 0.9987	0.9764	0.0503	25.3318	*a* = 0.7799 *k* = 0.02243 *b* = 1.002	0.9680	0.0599	35.8827
9	*a* = −2.267 *k* = 0.007768 g = 0.01015	0.9981	0.0137	1.8890	*a* = −11.28 *k* = 0.04076 g = 0.03812	0.9975	0.0165	2.7157	*a* = −10.25 *k* = 0.03782 g = 0.03526	0.9980	0.0147	2.1696	*a* = −3.118 *k* = 0.007031 g = 0.009621	0.9976	0.0166	2.7434
10	*a* = 0.9911 *k* = 0.007832 *n* = 1.186 *b* = −0.000238	0.9992	0.0088	0.7758	*a* = 0.9893 *k* = 0.006106 *n* = 1.294 *b* = −0.0002345	0.9991	0.0100	1.0029	*a* = 0.9902 *k* = 0.006428 *n* = 1.263 *b* = −0.0002225	0.9993	0.0087	0.7483	*a* = 0.993 *k* = 0.008024 *n* = 1.227 *b* = −0.0006588	0.9992	0.0096	0.9247

### 3.3 Color Parameters of Strawberry Slices

Table 4 provides the color values for both fresh strawberries and those subjected to drying through EHD, EHD‐hot air, and hot air methods. Remarkably, the fresh samples exhibited the highest L*, a*, and b* values, which experienced a reduction due to the influence of temperature. In a parallel observation to our research, Ozturk and Singh (2019) noted a decline in color parameters (L*, a*, and b*) during their study on hot air drying of strawberries. They attributed this phenomenon to factors like ascorbic acid oxidation, amino compound condensation, browning reactions, and carotenoid degradation. Upon examination of the table, a significant disparity in L* values (*p* < 0.05) was evident between the drying methods (EHD, EHD‐hot air, and hot air). Among the drying methods, the EHD method yielded samples with L* values that closely resembled those of the fresh samples, with the EHD‐hot air combined method and hot air method following in sequential order. Similar findings were reported by Hashinaga et al. (1999), who observed higher L* values in EHD‐dried apples compared with oven‐dried counterparts. Regarding a* values, the EHD‐hot air combined method yielded higher values than both the hot air and EHD methods. The a* values declined with temperature escalation in the hot air method and kV value reduction in the EHD applications. The b* values ranged from 25.6 ± 1.03 (fresh) to −11.69 ± 0.46 (55°C). With the exception of the 30 kV‐55°C treatment, there were no statistically significant differences observed in b* values among the various methods within the EHD‐hot air combination (*p* > 0.05). The Chroma (*C*) values, representing color saturation, demonstrated their highest expression in the EHD‐hot air combination method following the fresh product. The hue angle (*α*°) values of the fresh products exhibited a decrease ranging from 13.17% (30 kV) to 25.58% (55°C) as influenced by the applied drying process. The total color change (Δ*E*) value serves as an indicator of the degree of color alteration between fresh and dried products. A low Δ*E* value signifies better preservation of color parameters, indicating a closer resemblance to the fresh product (Siriamornpun, Kaisoon, and Meeso 2012). The hot air method exhibited the highest Δ*E* values, whereas the employment of the EHD method solely or in conjunction with hot air led to decreased Δ*E* values. A study by Esehaghbeygi and Karimi (2020) involving various methods for drying mint leaves found Δ*E* values of EHD‐dried samples to be lower than those dried in an oven, a correlation aligned with our findings.

**TABLE 4:** Color values of fresh and dried strawberry slices with different drying methods.

Drying conditions	Color parameters					
	L*	a*	b*	*C*	*α*°	*∆E*
Fresh	38.450 (0.532)^a^	36.274 (0.759)^a^	25.602 (1.031)^a^	44.400 (1.214)^a^	35.221 (0.517)^a^	—
EHD						
20 kV	36.238 (0.696)^b^	27.092 (0.190)^f^	15.490 (0.400)^e^	31.208 (0.411)^f^	29.770 (0.464)^c^	13.849 (0.504)^d^
30 kV	33.698 (0.177)^c^	28.894 (0.141)^e^	17.062 (0.123)^c^	33.555 (0.179)^e^	30.577 (0.091)^b^	12.246 (0.234)^b^
Hot Air						
50°C	29.428 (0.170)^f^	28.726 (0.222)^e^	16.140 (0.136)^d^	32.950 (0.255)^e^	29.345 (0.097)^c,d^	15.097 (0.275)^e^
55°C	24.928 (0.350)^g^	23.752 (0.242)^g^	11.690 (0.461)^f^	26.474 (0.392)^g^	26.211 (0.737)^f^	23.094 (0.483)^f^
EHD‐Hot Air						
20 kV‐50°C	31.680 (0.104)^d^	31.104 (0.132)^c^	17.054 (0.099)^c^	35.473 (0.140)^c^	28.750 (0.140)^e^	12.068 (0.121)^b^
20 kV‐55°C	30.594 (0.147)^e^	30.450 (0.095)^d^	16.536 (0.403)^c,d^	34.651 (0.378)^d^	28.515 (0.675)^e^	13.338 (0.303)^c,d^
30 kV‐50°C	31.298 (2.316)^d,e^	30.694 (0.526)^c,d^	16.956 (0.198)^c^	35.068 (0.259)^c,d^	28.937 (0.535)^d,e^	12.666 (1.199)^b,c^
30 kV‐55°C	30.528 (0.070)^e^	32.322 (0.135)^b^	18.514 (0.100)^b^	37.249 (0.145)^b^	29.819 (0.132)^c^	11.341 (0.135)^a^

*Note:* The statistics of each color parameter column have been applied separately, and the differences between the means with different letters in the same column are significant (*p* < 0.05).

### 3.4 Rehydration Capacity of Dried Strawberry Slices

**FIGURE 3:**
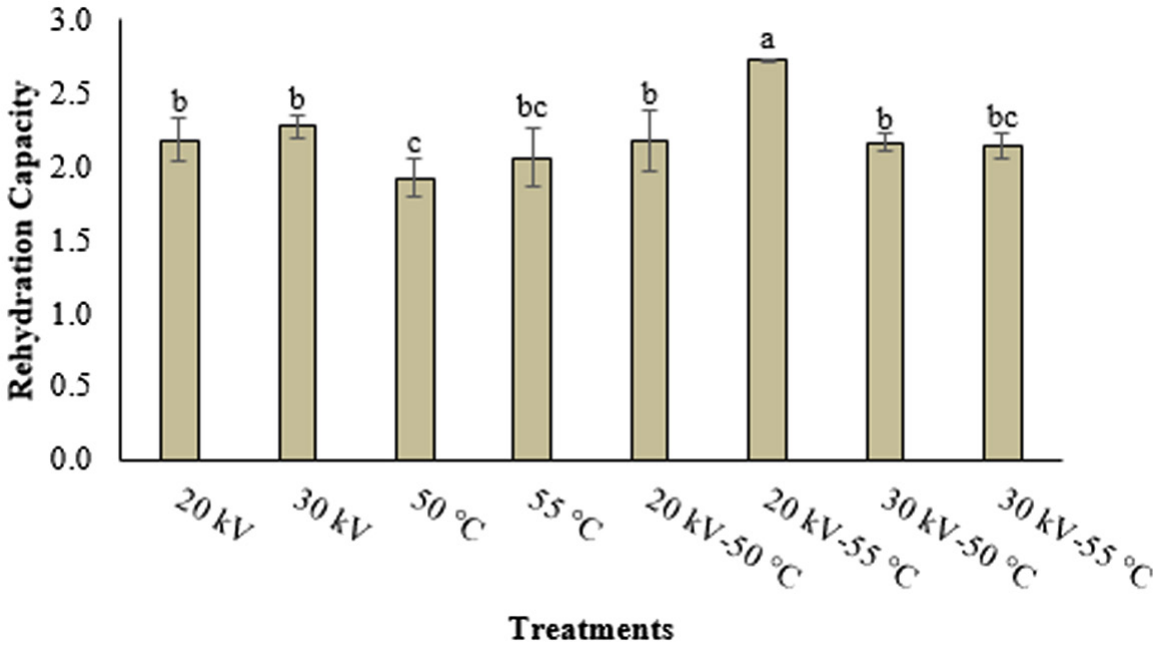
Rehydration capacity values of dried strawberry slices.

Typically employed prior to consuming dried foods, the rehydration process underscores the significance of rehydration properties, serving as markers for the physical and chemical changes occurring within dried products (Mujaffar and Lee Loy 2017). The rehydration capacity values of strawberry samples subjected to EHD, hot air, and EHD‐hot air combined methods are depicted in Figure 3. Among these methods, the highest rehydration value was recorded for the 20 kV‐55°C treatment; the most diminished values were discerned in samples subjected to drying at both 50°C and 55°C, as well as in those undergoing the 30 kV‐55°C treatment. Notably, neither the elevation of temperature within the hot air drying method nor the increment in voltage values in the EHD drying method exhibited a statistically significant impact on rehydration capacity (*p* > 0.05). This observation mirrors the findings of our study and aligns with research by Gamboa‐Santos et al. (2014), who reported that temperature elevation did not significantly influence the rehydration ratio in their strawberry drying investigation. Similarly, Dinani and Havet (2015) observed no statistical disparity in rehydration values for mushroom samples dried at high air velocity (2.2 m/s) when voltage values were augmented, in line with our study's outcomes.

### 3.5 Microstructure of Dried Strawberry Slices

**FIGURE 4:**
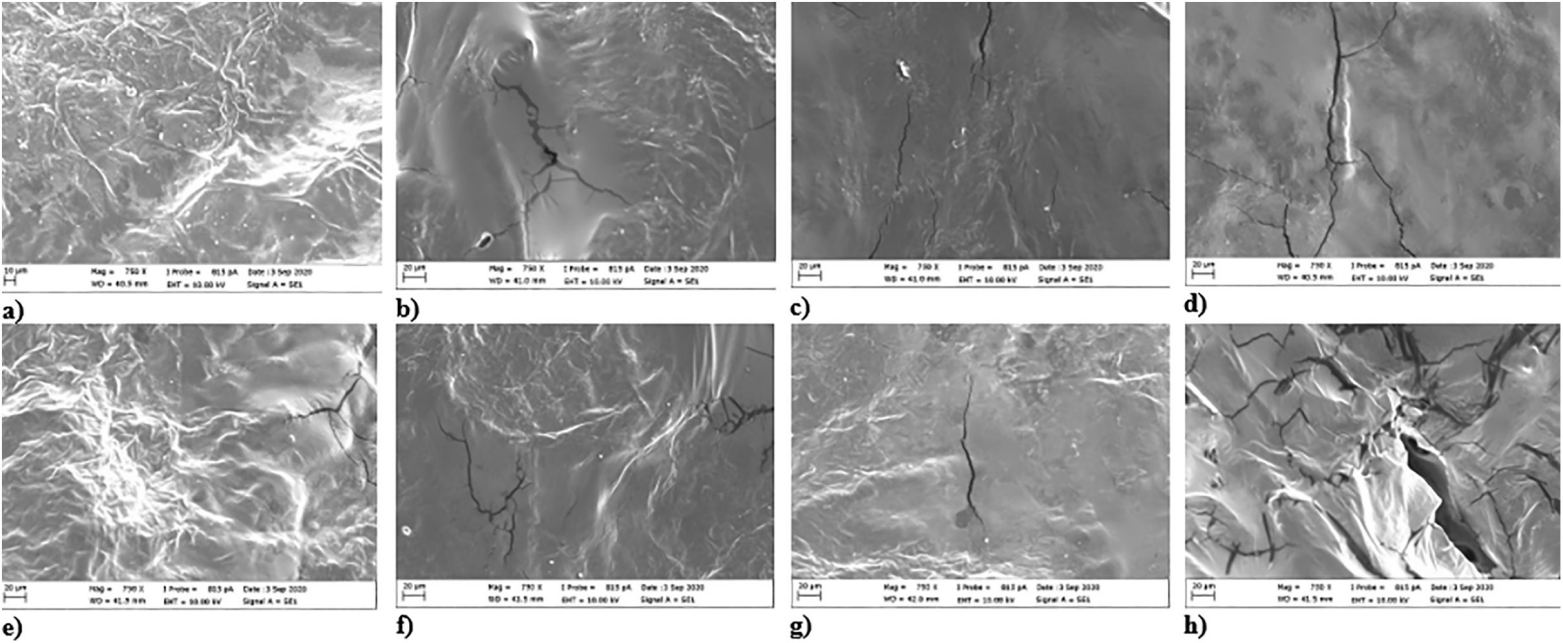
SEM images of strawberry slices dried under different drying conditions: (a) 20 kV, (b) 30 kV, (c) 50°C, (d) 55°C, (e) 20 kV‐50°C, (f) 20 kV‐55°C, (g) 30 kV‐50°C, and (h) 30 kV‐55°C.

Scanning electron microscope images illustrating the surface structures of strawberry samples subjected to three distinct drying methods (EHD, EHD‐hot air, and hot air), have been presented in Figure 4. Notably, the samples dried using 20 kV revealed the presence of small crystal structures on their surfaces. However, in other methods, the crystals diminished due to the influence of temperature. Within samples dried through hot air and EHD‐hot air methods, a hardening of the tissue surface was observed. Excessive hardening induced by elevated temperatures led to the formation of cracks on the product's surface, further exacerbated by heightened stress. Furthermore, an escalation in temperature resulted in an increased number of fractures (Bai et al. 2002; Zielinska, Sadowski, and Błaszczak 2016). Remarkably, within the hot air drying method, the surface structures of the samples lost their three‐dimensional quality, appearing flatter compared with other methods. Conversely, within the EHD method, a three‐dimensional structure was discernible in the samples dried using 20 kV. However, for the samples dried at 30 kV, the surface structure appeared flatter in comparison with those dried at 20 kV, forming a structure marked by fractures and voids. With an increase in voltage value, the incidence of breakage is amplified (Yu et al. 2018).

### 3.6 Total Soluble Solids and pH Values of Strawberry Slices

The presented data in Table 5 furnishes the pH and total soluble solid (TSS) measurements of strawberry specimens subjected to various drying techniques. Upon scrutinizing Table 5, it becomes evident that the electric potential applied in the EHD method and the temperature employed in the hot air drying method do not exert a statistically significant influence on the parameters under consideration (*p* > 0.05). These parameters represent pivotal indicators of the resultant quality of the dehydrated products. The most substantial concentrations of soluble solids are manifest in the specimens that underwent the drying process at either 20 kV and 50°C or 30 kV and 50°C. In a prior investigation conducted by Fang et al. (2009), it was noted that the soluble solids content in the fresh produce was comparatively lower, whereas no statistically meaningful discrepancies in soluble solids content were observed among products subjected to distinct drying temperatures. These findings align with the outcomes of our own research.

**TABLE 5:** TSS and pH values of strawberry slices.

Product	TSS	pH
Fresh	9.67 ± 0.47 d	3.42 ± 0.07 c
20 kV	63.40 ± 1.25 b	3.45 ± 0.08 b,c
30 kV	60.60 ± 3.17 b,c	3.43 ± 0.09 c
50°C	61.80 ± 5.13 b,c	3.50 ± 0.14 a,b
55°C	57.80 ± 3.30 c	3.59 ± 0.09 a
20 kV‐50°C	60.40 ± 1.93 b,c	3.43 ± 0.09 c
20 kV‐55°C	69.00 ± 2.08 a	3.49 ± 0.04 a,b,c
30 kV‐50°C	71.00 ± 1.25 a	3.42 ± 0.06 c
30 kV‐55°C	63.40 ± 4.42 b	3.53 ± 0.02 a,b,c

*Note:* The statistics of Brix and pH column have been applied separately, and the differences between the means with different letters in the same column are significant (p < 0.05).

It was ascertained that the variations in electric potential and temperature, as parameterized within the different drying methodologies, yield no statistically significant alterations in the pH values of the resultant products (*p* > 0.05). It is pertinent to note that the pH value of strawberry specimens subjected to hot air drying surpasses that of the fresh product. In a parallel study, Vega‐Gálvez et al. (2009) observed an analogous trend in the pH values of fresh produce, which augmented with increasing drying temperatures in the context of their examination of red pepper subjected to hot air drying.

## 4 Conclusion

This study encompassed an analysis of the drying behavior, modeling, color attributes, rehydration capacity, microstructural properties, TSS, and pH values of strawberry slices across various drying conditions Drying times varied, with the longest duration of 530 min observed for the 20 kV application and the shortest, 110 min, for the 30 kV‐55°C application. The moisture ratio extracted from the product during drying was subjected to thin‐layer drying models, revealing the Midilli et al. model as the most accurate predictor for EHD and hot air drying conditions, whereas the Wang and Singh model fit best for EHD‐hot air combined drying conditions. An evident reduction in b* values was noted under the influence of different drying conditions. Notably, the EHD‐dried samples exhibited lower Δ*E* values compared with other methods. With the exception of the 20 kV‐55°C application, the rehydration capacities of strawberry samples subjected to the EHD‐hot air combination drying method did not significantly differ. Predominant cracks on the product surface were evident in samples subjected to the 30 kV‐55°C application. The highest TSS values were determined in dried strawberry samples obtained from 20 kV‐55°C and 30 kV‐50°C treatments. The change of air temperature in the hot air method and the change of volt value in the EHD method did not statistically affect the pH value (*p* > 0.05). This study contributes valuable evidence demonstrating that the EHD method yields high‐quality dried strawberry products. Moreover, it is anticipated that integrating the EHD method with hot air for strawberry drying could offer time‐saving advantages compared with employing either method individually, particularly due to the extended drying time associated with EHD and hot air drying. Furthermore, the study's findings can foster the progression of future research and establish an information foundation for the development of industrial‐scale drying devices.

## Author Contributions

**Ahmet Polat:** conceptualization (lead), data curation (lead), formal analysis (lead), funding acquisition (lead), investigation (lead), methodology (lead), software (lead), supervision (lead), validation (lead), visualization (lead), writing – original draft (lead), writing – review and editing (lead).

## Ethics Statement

The author has nothing to report.

## Conflicts of Interest

The author declares no conflicts of interest.

## Data Availability

The author has nothing to report.
